# Supplementary material for: The causal relationship between the human gut microbiota and pyogenic arthritis: a Mendelian randomization study
Source: Front Cell Infect Microbiol. 2024 Nov 26;14:1452480. doi: 10.3389/fcimb.2024.1452480 (PMC11629706; doi:10.3389/fcimb.2024.1452480)
Supplement: Supplementary file 1 [file DataSheet1.zip › mendelian test/ebi-a-GCST90027848.csv_leave_one_out.pdf]

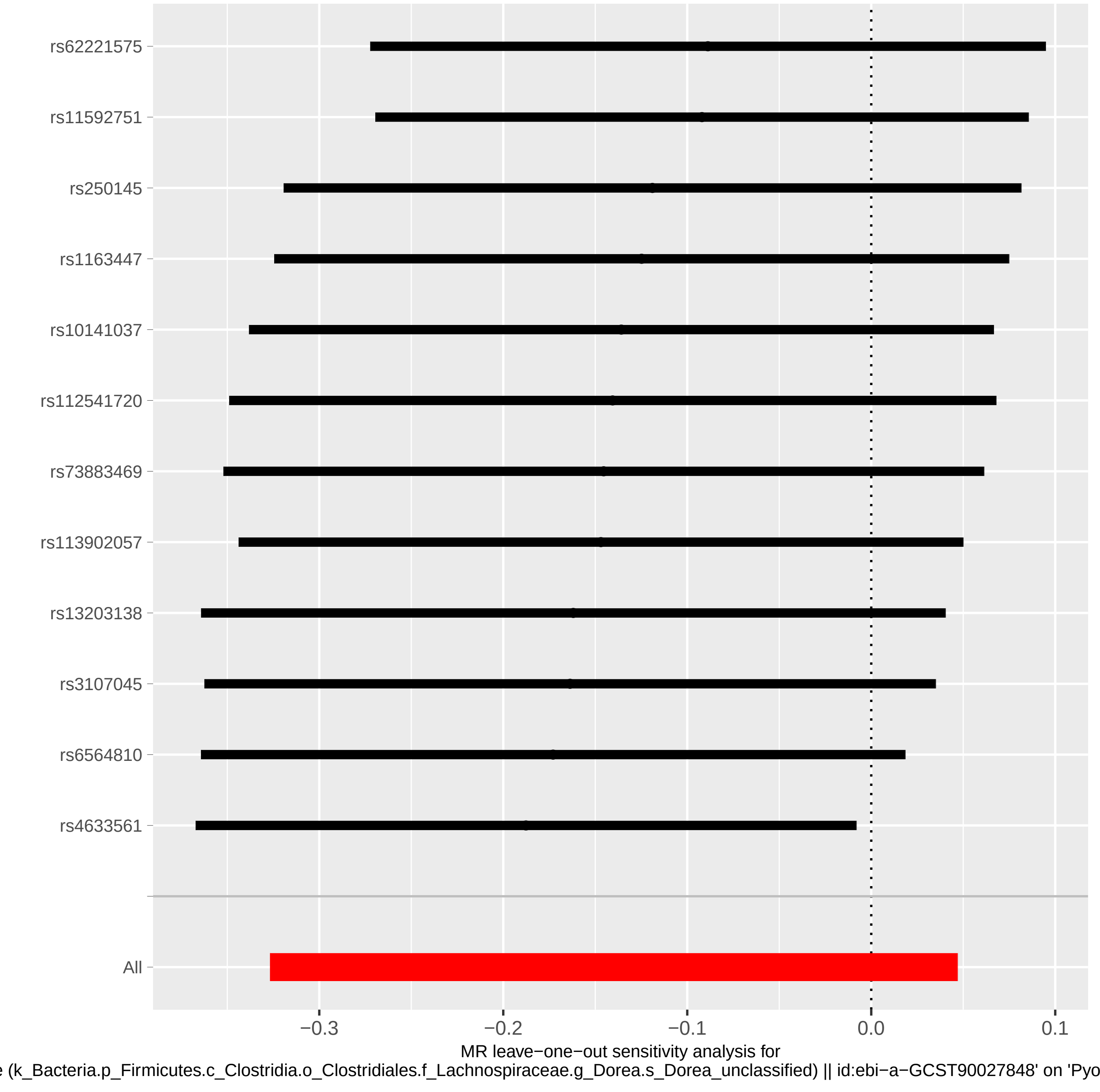
rs62221575
rs11592751
rs250145
rs1163447
rs10141037
rs112541720
rs73883469
rs113902057
rs13203138
rs3107045
rs6564810
rs4633561
All
−0.3
−0.2
−0.1
0.0
0.1
MR leave−one−out sensitivity analysis for
e (k_Bacteria.p_Firmicutes.c_Clostridia.o_Clostridiales.f_Lachnospiraceae.g_Dorea.s_Dorea_unclassified) || id:ebi−a−GCST90027848' on 'Pyo
